# Postoperative rehabilitation in a patient with Proteus syndrome following scoliosis surgery: A case report

**DOI:** 10.1097/MD.0000000000045728

**Published:** 2025-10-31

**Authors:** Houyi Zhu, Huijuan Liu, Xinglou Li, Ningning Zhao, Yongbo Teng, Ran Shi

**Affiliations:** a Department of Rehabilitation Medicine, The First Affiliated Hospital of Shandong First Medical University & Shandong Provincial Qianfoshan Hospital, Jinan, China; b Research Center for Child Health, Key Laboratory of Birth Regulation and Control Technology of National Health Commission of China, Shandong Provincial Maternal and Child Health Care Hospital Affiliated to Qingdao University, Jinan, China.

**Keywords:** AKT1, case report, proteus syndrome, rehabilitation, scoliosis

## Abstract

**Rationale:**

Proteus syndrome (PS) is a rare congenital disorder characterized by dysregulated overgrowth and an array of complex skeletal anomalies, including scoliosis. The rehabilitation process following spinal surgery has never been reported.

**Patient concerns:**

A 10-year-and-3-month-old patient with PS caused by an serine/threonine protein kinase 1 (AKT1) gene mutation, who was referred to our hospital for bilateral lower extremity weakness following scoliosis surgery.

**Diagnoses:**

Proteus syndrome.

**Interventions:**

The patient received a comprehensive rehabilitation program, including lower limb muscle strength training, passive-active joint mobilization, and joint manipulation, in conjunction with electronic biofeedback therapy and acupuncture, administered by a multidisciplinary rehabilitation team.

**Outcomes:**

The patient’s lower limb mobility has improved compared to the initial presentation upon admission, and no complications have been observed.

**Lessons:**

The rehabilitation program presented in this study has demonstrated significant efficacy in mitigating muscle weakness in PS patients following surgical intervention for scoliosis.

## 1. Introduction

Proteus syndrome (PS) (online Mendelian inheritance in man #176920) is a sporadic and complex congenital disorder first described by Cohen et al in 1979.^[1,2]^ The male-to-female ratio is approximately 1.9:1, with an estimated incidence of <1 in 1,000,000.^[3]^ To date, only around 200 cases have been documented globally. The pathogenesis of this condition is characterized by chimeric mutations in the serine/threonine protein kinase 1 (AKT1) gene located on chromosome 14 in somatic cells, which lead to dysregulated cellular proliferation.^[4]^ PS manifests with significant phenotypic variability, presenting as asymmetric and irregular overgrowth that can involve multiple tissues, including skeletal and adipose tissues, connective tissues, skin, and the central nervous system, with skeletal and adipose tissues being the most commonly affected. The internationally recognized diagnostic criteria are complex and often overlap with other growth-related disorders, leading to potential misdiagnosis. The gold standard for diagnosis is genetic testing, with next-generation sequencing technology serving as a crucial tool for the diagnosis of rare cases.^[5]^ Accurate diagnosis necessitates a comprehensive evaluation that includes the patient’s clinical symptoms, physical examination findings, histopathological results, and genetic testing. Currently, there is no universally accepted treatment protocol for this condition; management primarily focuses on surgical interventions aimed at correcting deformities to enhance the patient’s quality of life. Additionally, targeted therapies such as the AKT inhibitor Miransertib^[6]^ and growth factor receptor antagonists^[7]^ have emerged as significant areas of research. Patients with PS are at heightened risk of mortality due to complications such as deep vein thrombosis and pulmonary embolism; therefore, early diagnosis and timely intervention are critical for improving patient outcomes.

Surgical intervention is necessitated when skeletal lesions adversely affect physiological function. PS is a clinically rare disorder characterized by complex and variable manifestations. Scoliosis is a common condition exhibiting significant variability and specificity^[8]^; however, there is limited literature on the subject, and the effectiveness of rehabilitation therapy following surgical treatment for scoliosis has not been reported in the literature. This article reports a case of postoperative rehabilitation for a patient with PS caused by an AKT1 gene mutation, aiming to enhance clinicians’ comprehension of the diagnostic and therapeutic approaches to this condition.

## 2. Case presentation

### 2.1. Basic information about the patient

**Figure 1.**
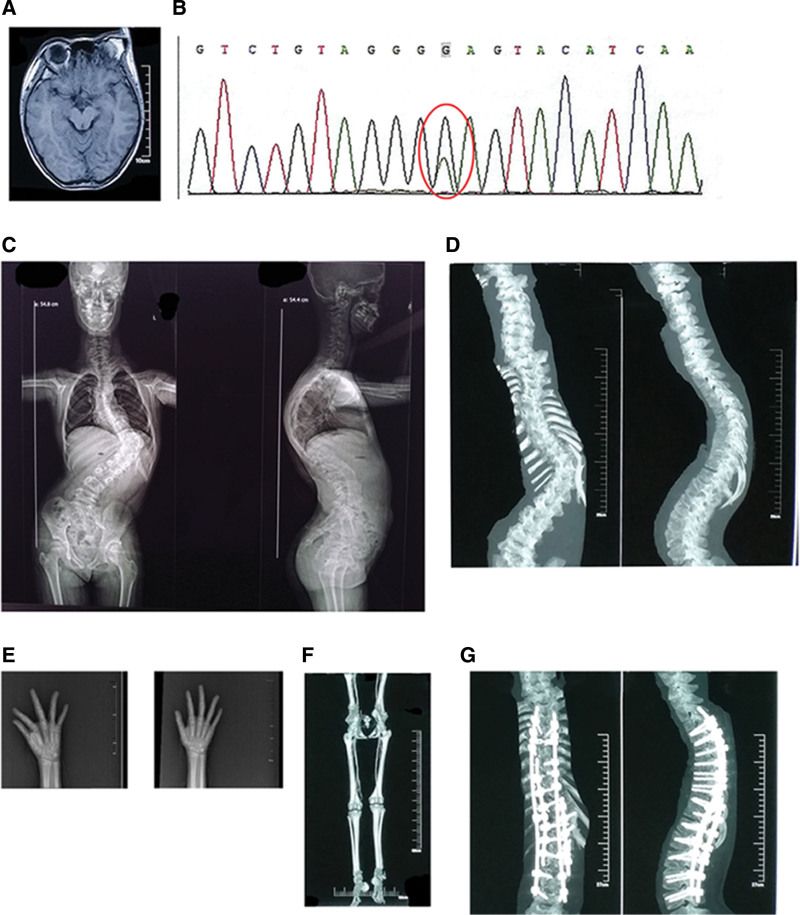
Patient case data. (A) Frontal bone prominence at the superior margin of the left eyebrow. (B) Identification of the AKT1 gene c.G49A mosaic mutation in the patient’s skin tissue and urine sediment. (C) Anteroposterior and lateral full-spine X-ray images illustrating the presence of spinal scoliosis. (D) Three-dimensional reconstruction of spinal CT revealing significant scoliosis and kyphotic deformities, alongside developmental abnormalities in several lumbar vertebrae. (E) Anteroposterior and oblique X-ray views of both hands demonstrating enlargement of the proximal phalanges of the right third and fourth fingers. (F) CT angiography of the lower extremities displaying malformations of the left femoral artery and a patchy low-density lesion in the right calcaneus. (G) Postoperative three-dimensional CT reconstruction indicating satisfactory spinal correction. AKT1 = serine/threonine protein kinase 1, CT = computed tomography.

The patient is a 10-year-and-3-month-old girl who presented with bilateral lower limb weakness occurring one month after scoliosis correction surgery. Her parents are not closely related, and she is a G1P1, having been born at term via spontaneous vaginal delivery. The mother reported no history of illness or medication during pregnancy and no episodes of asphyxia. At birth, the patient weighed 3.4 kg (P50-75) and measured 50 cm in length (P50-75), with no noted limb abnormalities. At 18 months of age, a mass was identified above the left eyebrow (Fig. 1A), which was subsequently followed by the development of café-au-lait spots on the skin. At 2 years old, the patient exhibited a waddling gait, and by age 6, a limping gait was observed. X-rays revealed a 2 cm shortening of the right lower limb. By age 7, asymmetries in the thoracolumbar region were documented, and at 9.5 years, ultra-deep sequencing identified a low-frequency mosaic mutation in the AKT1 gene (c.49G > A, with a nucleotide alteration from G to A at the 49th position of the coding region) in the patient’s skin tissue and urine sediment (Fig. 1B). This variant was not detected in the proband’s peripheral blood or oral mucosa, indicating a de novo mutation and leading to a diagnosis of PS.

In the preceding 4 years, the patient experienced progressive worsening of thoracolumbar asymmetry. Standing full-spine X-rays demonstrated scoliosis with a Cobb angle of 96° in the thoracolumbar region (Fig. 1C). Full-spine computed tomography (CT) and three-dimensional reconstruction revealed severe scoliosis and kyphotic deformities, along with developmental anomalies in some lumbar vertebrae (Fig. 1D). Anteroposterior and oblique X-rays of both hands indicated enlargement of the proximal phalanges of the right third and fourth fingers (Fig. 1E). CT angiography of the lower limbs showed multiple tortuous vessels in the distal segment of the left femoral artery, with visualization of the left femoral vein and a mass superior to the left patella, accompanied by adjacent bone irregularities. The right lower limb exhibited superficial venous varicosities, and a patchy low-density lesion was noted in the right calcaneus (Fig. 1F). The electrocardiogram and echocardiogram results were unremarkable, and routine laboratory tests – including complete blood count, electrolyte levels, liver and kidney function tests, and lipid profiles – revealed no abnormalities. Coagulation function tests indicated decreased activity of coagulation factors II (67.5%), VIII (77.2%), IX (67.0%), X (71.8%), and XI (67.1%), with protein S activity also reduced to 53.1%. Spinal correction was satisfactory, as postoperative CT with three-dimensional reconstruction demonstrated proper alignment and stable internal fixation, with no signs of loosening or fracture (Fig. 1G). Twenty-four hours post-surgery, the child exhibited weakness in both lower extremities and subsequently presented to our hospital for consultation after a month of at-home convalescence following discharge.

Physical examination: Height: 140.5 cm (P25–P50); Weight: 32.3 kg (P25–P50). The skin examination revealed no abnormalities in hair distribution but showed multiple café-au-lait macules. A bony prominence measuring 2 × 1.5 cm was noted above the left eyebrow, and asymmetrical lipomatous swellings were observed on both plantar surfaces. Additionally, an irregular 3 × 3 cm cerebriform connective tissue nevus was present on the right sole. A longitudinal surgical scar, approximately 38 cm in length, was visible along the midline of the spine; the incision was well-healed, and there was no tenderness upon palpation. Both abdominal reflex and bilateral patellar reflexes were present, with no pathological reflexes elicited. The lower limb muscles exhibit no signs of hypertrophy.

Specialist assessment: Muscle tone (Modified Ashworth Scale^[9]^): The right upper limb exhibited normal muscle tone, while the left upper limb and both lower limbs showed decreased muscle tone. Muscle strength (manual muscle testing – MMT): Right side: Elbow flexion 5/5, wrist extension 5/5, elbow extension 5/5, middle finger flexion 5/5, little finger abduction 5/5, hip flexion 0/5, knee extension 4/5, ankle dorsiflexion 4/5, toe dorsiflexion 4/5, ankle plantarflexion 4/5. Left side: Elbow flexion 4/5, wrist extension 4/5, elbow extension 4/5, middle finger flexion 4/5, little finger abduction 4/5, hip flexion 4/5, knee extension 4/5, ankle dorsiflexion 4/5, toe dorsiflexion 4/5, ankle plantarflexion 4/5. Joint range of motion: Passive range of motion was within normal limits. Circumference: No significant discrepancies were observed bilaterally. Pain (visual analog scale – VAS): The patient reported a pain score of 0, indicating no pain. Sensation: Superficial sensation: The right side demonstrated intact superficial sensation, including the occipital condyle, acromion, lateral aspect of the cubital fossa, and the skin over the dorsal surfaces of the thumb, middle finger, and little finger, with normal sensation in the axillary midpoint, supraclavicular fossa, and the third to fifth intercostal spaces. Sensation was diminished at T4 and below. The left side exhibited similar results, with intact superficial sensation in the aforementioned areas and diminished sensation in the third to fifth intercostal spaces at T4 and below. Deep sensation: Deep sensation was preserved. Hearing, vision, and cognitive function were all assessed to be within normal limits.

Auxiliary examinations: Routine analyses of blood, urine, and stool, as well as assessments of liver function, kidney function, electrolyte levels, blood glucose, lipid profiles, and coagulation parameters, demonstrated no significant abnormalities. Echocardiography and electrocardiogram findings were within normal limits. Non-contrast CT imaging of the head revealed localized bone expansion in the left orbital region and the frontal bone.

Activities of daily living were evaluated using the Modified barthel index: Bladder control: 10 points, Bowel control: 10 points, Dressing: 5 points, Mobility: 10 points, Feeding: 10 points, Transfer: 5 points, Toileting: 5 points, Stairs: 0 points, Bathing: 0 points, Grooming: 0 points. The total score of 55 points indicates a moderate degree of impairment in activities of daily living.

### 2.2. Rehabilitation treatment

The patient underwent a comprehensive rehabilitation program provided by a multidisciplinary team consisting of a physiatrist, physical therapist, occupational therapist, rehabilitation nurse specialist, dietitian, and medical social worker. The therapists provided individualized instruction in lower limb strength training, passive-active joint mobilization, and joint stabilization exercises, supplemented by localized massage and manual therapy techniques. Additional modalities included electronic biofeedback therapy and acupuncture. Rehabilitation sessions were conducted twice daily, each lasting 30 minutes. Pharmaceutical interventions consisted of mecobalamin and nerve growth factor to promote neuroprotection, as well as sodium saponin to mitigate neuronal edema. The treatment approach prioritized simple, easily achievable exercises with minimal risk of adverse effects. The patient was guided in practicing activities of daily living, such as dressing, feeding, and personal hygiene, as well as training in bed mobility. Moreover, psychological support and counseling were actively provided to both the patient and their family to bolster the patient’s self-confidence and adherence to the therapeutic regimen. The therapeutic course was uneventful, with no adverse reactions or untoward incidents observed.

### 2.3. Rehabilitation outcomes

**Figure 2.**
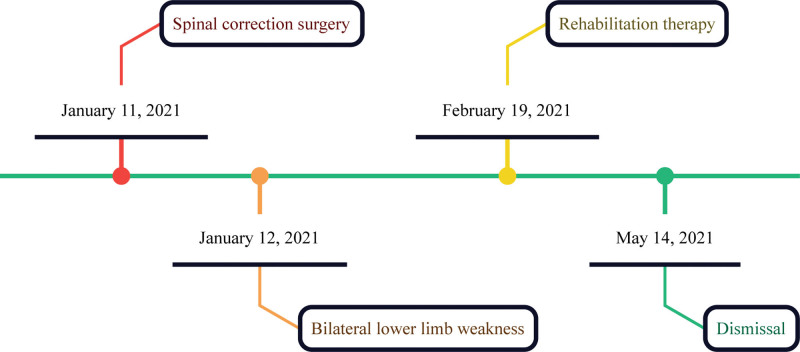
Timeline of the treatment process.

Following 12 weeks of inpatient rehabilitation, the patient exhibited marked improvement in lower limb function compared to the pre-admission baseline, with no complications recorded. Notably, there were deformities observed in both toes; however, no edema was present in the lower limbs. Right-sided superficial sensation in the third to fifth intercostal spaces remained intact, while sensitivity was diminished at the T4 vertebral level and below. On the left side, the third to fifth intercostal spaces were also normal, with similar reductions noted at T4 and below. The activities of daily living (modified barthel index) assessment yielded the following scores: Bladder control: 10 points, Bowel control: 10 points, Dressing: 5 points, Mobility: 10 points, Feeding: 10 points, Transfer: 10 points, Toileting: 5 points, Stairs: 5 points, Bathing: 0 points, Grooming: 0 points, resulting in a cumulative score of 65 points. The modified barthel index score improved by 10 points, indicating some improvement in activities of daily living, and the patient was successfully discharged (Fig. 2). The patient actively participated in the treatment and found the rehabilitation sessions helpful. She noted significant improvements in mobility and daily functioning, which enhanced her overall well-being.

## 3. Discussion

PS is a rare disorder characterized by asymmetric and progressive abnormal tissue overgrowth, classified as multilayered dysplastic overgrowth syndrome. The clinical manifestations of PS are notably complex and heterogeneous. In 1989, Samlaska et al^[10]^ summarized the clinical features observed in 34 cases of PS and proposed revisions to the diagnostic criteria. However, due to the considerable variability in clinical presentations, Biesecker et al^[11]^ established more specific diagnostic criteria in 1999, which encompassed both general and specific standards. Subsequently, in 2004, Turner et al^[12]^ refined these criteria to formulate diagnostic, differential diagnostic, and assessment standards for PS. Their framework includes 3 general categories and 3 specific categories (Table 1).

**Table 1:** Revised diagnostic criteria for Proteus syndrome.

Category	Criteria*	Specific details
General criteria	Must be met entirely	1. Mosaic distribution of lesions
		2. Progressive course
		3. Sporadic occurrence
Specific criteria categories		
Category A	Cerebriform connective tissue nevus	Characterized by deep grooves and gyrations as seen on the surface of the brain
Category B	1. Linear epidermal nevus	–
	2. Asymmetric, disproportionate overgrowth	one or more a. Limbs: - Arms/legs - Hands/feet/digits - Extremities b. Hyperostosis of the skull c. External auditory meatus d. Megaspondylodysplasia e. Viscera: - Spleen/thymus
	3. Specific tumors before the second decade	One of the following: - Ovarian cystadenoma - Parotid monomorphic adenoma
Category C	1. Dysregulated adipose tissue	Either one: a. Lipomas b. Regional absence of fat
	2. Vascular malformations	One or more: a. Capillary malformation b. Venous malformation c. Lymphatic malformatio
	4. Lung cysts	–
	5. Facial phenotype	All: a. Dolichocephaly b. Long face c. Down slanting palpebral fissures and/or minor ptosis d. Low nasal bridge e. Wide or anteverted nares f. Open mouth at rest

* A definitive diagnosis of PS requires that the patient meets all general criteria, as well as at least one criterion from category A, or 2 criteria from category B, or 3 criteria from category C.

For a definitive diagnosis of PS, cases must meet all 3 major criteria and exhibit either one A category criterion or two B category criteria, or alternatively, three C category criteria. In the present case, the diagnosis was established based on the patient’s fulfillment of all 3 general criteria alongside 3 specific criteria: namely, brain-like connective tissue nevi (A category), finger asymmetry with disproportionate overgrowth (B category), and vascular malformations (C category). Furthermore, an AKT1 mutation was identified in both the patient’s skin tissue and urinary sediment, thereby corroborating the diagnosis of PS.

Somatic activating mutations of the AKT gene are detected in the majority of PS patients and have been linked to excessive growth in skin, bone, connective tissue, brain, and other organ systems. These mutations also play a significant role in the development of benign tumors associated with this condition.^[13]^

Effective treatment options for PS remain limited. Management primarily focuses on symptomatic treatment aimed at enhancing patients’ quality of life and alleviating the burdens faced by them and their families.^[14]^ Given that both PS and scoliosis are associated with physical limitations and diminished self-esteem, early diagnosis and timely intervention are crucial. Surgical intervention for scoliosis in PS patients is complex and may require various osteotomies for adequate deformity correction. In this case, the patient presented with a significant spinal deformity, with a Cobb angle of approximately 96° in the thoracolumbar region and pronounced kyphosis. The orthopedic team performed posterior thoracolumbar fusion and segmental pedicle screw osteotomy (SPO) from T7 to L2, resulting in satisfactory spinal alignment correction, consistent with prior studies.^[8,15]^

However, one month postoperatively, the patient exhibited weakness in both lower limbs. After 12 weeks of multidisciplinary rehabilitation, the barthel index improved by 10 points, reflecting enhanced functional abilities. While rehabilitation training significantly improves daily living activities,^[16–19]^ literature addressing postoperative rehabilitation following scoliosis correction in PS patients is lacking. Our findings provide new insights into the comprehensive management of individuals with PS.

The identification of AKT1 mutations has provided valuable insights and theoretical foundations for the diagnosis and treatment of PS, as well as new opportunities for drug development. Miransertib is a potent inhibitor of AKT isoforms 1, 2, and 3, which has primarily been evaluated in adult oncology trials.^[20]^ Keppler-Noreuil et al conducted a non-randomized clinical trial that demonstrated Miransertib’s ability to reduce AKT phosphorylation Keppler-Noreuil.^[21]^ In 2020, Biesecker et al^[6]^ reported the application of Miransertib in a PS patient, noting that the treatment resulted in pain relief and a deceleration of the progression of plantar cortical-like connective tissue lesions. It is noteworthy that the minimum age of participants in these drug trials was 12 years; consequently, the treatment is not currently applicable to the patient reported in this study, who is only 10 years and 3 months old.

## 4. Conclusion

For patients with PS who develop scoliosis, in addition to active symptomatic treatment, comprehensive rehabilitation therapy has demonstrated significant efficacy in alleviating postoperative bilateral lower-limb muscle weakness. This dual approach not only enhances patients’ abilities to engage in daily activities and fosters their social participation but also significantly improves their overall quality of life. Future research should focus on further elucidating the pathogenesis of this syndrome, refining the rehabilitation assessment framework, and strengthening psychological interventions. These efforts aim to optimize rehabilitation outcomes and provide comprehensive support for patients.

## Acknowledgments

We would like to extend my sincere gratitude for the financial support received from the Shandong Research Hospital Association Research Fund and the Key Discipline Construction Project of Traditional Chinese Medicine in Shandong Province.

## Author contributions

**Conceptualization:** Houyi Zhu, Huijuan Liu, Ran Shi.

**Data curation:** Xinglou Li.

**Formal analysis:** Yongbo Teng.

**Visualization:** Ningning Zhao.

**Writing – original draft:** Houyi Zhu, Huijuan Liu.

**Writing – review & editing:** Ran Shi.
